# Comment on Gratwicke et al. Nutritional Interventions to Improve Sleep in Team-Sport Athletes: A Narrative Review. *Nutrients* 2021, *13*, 1586

**DOI:** 10.3390/nu13093103

**Published:** 2021-09-03

**Authors:** Ian C. Dunican, Jennifer H. Walsh

**Affiliations:** Centre for Sleep Science, School of Human Sciences, The University of Western Australia, Crawley, WA 6009, Australia; jennifer.walsh@uwa.edu.au

We recently read with great interest the recent paper by Gratwicke, M et al. (2021), “Nutritional Interventions to Improve Sleep in Team-Sport Athletes: A Narrative Review”, within the journal *Nutrients* [1]. We applaud the research team for attempting to highlight the interaction between chrononutrition and sleep. However, we believe the authors may have overlooked some vital evidence, included some erroneous details and overstated the practical applications of their review, which could be potentially misleading.

The authors’ stated aim was to review nutritional strategies that can enhance sleep quality and quantity in athletes (not team-sport athletes as specified in the title). However, what defines ‘good’, ‘improved’ or ‘ideal’ sleep quantity and sleep quality are unclear in the current sleep-related literature [2], and the authors have not clarified their definition.

In their discussion, the authors state that “While promoting recovery is beneficial to athletes, tart cherry juice has enhanced sleep indices as assessed by PSG and wrist actigraphy monitoring in healthy individuals without the presence of sleep problems [3], and individuals with sleep problems such as insomnia [4].” This statement exaggerates the quality of evidence generated from these studies (which are also referenced incorrectly in the text) as neither has utilized the gold standard polysomnography (PSG) to assess sleep. Indeed, the quality of evidence of many of the studies included in the review is low, especially those with low sample sizes, no control condition, no randomization or no objective measures of sleep. It is appreciated that the authors cannot alter the quality of the studies reviewed. However, such limitations should be highlighted to ensure readers are able to interpret the quality of the evidence presented. Furthermore, practical recommendations should not include findings from studies with weak evidence. The strongest evidence available regarding impact of nutritional interventions on sleep relates to caffeine [5] and alcohol [6,7], evidence for which was not presented or discussed.

The authors also note that “*Folate deficiency has been linked to insomnia and restless leg syndrome*” [8,9]. However, to the best of our knowledge, the link between insomnia, restless leg syndrome and folate deficiency is not apparent in the studies referenced or any studies other than a single pregnant population [10].

In conclusion, we believe these errors, omissions and interpretations are potentially misleading to future readers, researchers and practitioners in this field and should be noted in conjunction with the manuscript.
